# A novel role of the splenic volume in Crohn’s disease: evaluating the efficacy of infliximab

**DOI:** 10.3389/fphar.2023.1246657

**Published:** 2023-08-17

**Authors:** Xuan Shi, Jia-Hui Wang, Sheng-Xiang Rao, Tao-Tao Liu, Hao Wu

**Affiliations:** ^1^ Department of Gastroenterology and Hepatology, Zhongshan Hospital, Fudan University, Shanghai, China; ^2^ Department of Radiology, Zhongshan Hospital, Fudan University, Shanghai, China

**Keywords:** splenic volume, infliximab, Crohn’s disease, computed tomography, drug efficacy

## Abstract

**Background:** A number of patients with Crohn’s disease (CD) suffer from loss of response to infliximab (IFX) therapy. Splenic volume is reported to be enlarged in patients with CD compared to normal individuals. The association between splenic volume and IFX efficacy in CD remains unclear.

**Methods:** We performed a retrospective study of patients with CD who received regular IFX treatment at Zhongshan Hospital, Fudan University, between August 2015 and December 2021. We collected baseline characteristics and clinical features from medical records in the CD database of Zhongshan Hospital. We accurately measured the splenic volume using semi-auto spleen segmentation software, followed by the analysis of splenic volume and IFX efficacy.

**Results:** We included 49 patients with CD receiving IFX treatment, of whom 41 responded to IFX and 8 failed to respond to IFX. Splenic volume, as well as volume adjusted for body mass index (SV/BMI) and body weight (SV/W), was significantly decreased after IFX treatment in responders but increased in non-responders compared to the volume before the treatment. Accordingly, the levels of leukocyte count, platelet count, C-reactive protein (CRP), and erythrocyte sedimentation rate (ESR) were decreased after IFX treatment in responders. Contrarily, the levels of hemoglobin, albumin, and tumor necrosis factor (TNF)-α were elevated in responders. Moreover, both CRP and TNF-α levels were significantly positively correlated with SV/BMI in all patients.

**Conclusion:** Splenic volume, especially SV/BMI and SV/W, was reduced after IFX treatment in CD patients responsive to IFX. SV/BMI was positively correlated with disease activity. Splenic volume is a promising indicator to evaluate IFX efficacy in CD.

## 1 Introduction

Crohn’s disease (CD), as a main subtype of inflammatory bowel disease (IBD), is a complex chronic granulomatous inflammatory disease of the gut. CD is characterized by an extensive range of lesions and a tendency to relapse throughout life, leading patients to suffering. Recently, great therapeutic advances in biological agents have resulted in the induction and maintenance of disease remission, especially for patients with moderate to severe CD (Li et al., 2021; Hong et al., 2022). Biologicals comprising multiple agents aimed at various molecular targets include infliximab (IFX), a monoclonal antibody to tumor necrosis factor (TNF)-α (Buhl et al., 2017). Unfortunately, the annual risk for loss of response to IFX accounts for more than 10% per patient-year in CD patients receiving standard therapy (Qiu et al., 2017), which leads to disease progression and economic loss. Thus, accurate assessment of IFX therapeutic response remains a challenge in CD patient management.

The interaction between T cells and antigen-presenting cells and the participation of various inflammatory cytokines are critical to the pathogenesis of CD (Baumgart and Carding, 2007). As a crucial lymphoid organ in the immune system, the spleen may be influenced by the inflammation triggered by CD progression. Splenomegaly was observed in various conditions, including liver disease, portal hypertension, heart disease, blood disease, infection, and cancer. Previous studies also reported splenomegaly in CD (Rozen et al., 1977; Palmer et al., 1981; Pereira et al., 1987). Additionally, Khashper et al. (2022) and Kawashima et al. (2022) discovered that splenic volume and spleen volume adjusted for body weight were significantly increased in CD patients compared to the healthy population through a retrospective study. Moreover, both splenic volume adjusted for body mass index (BMI) and splenic volume adjusted for body weight, rather than splenic volume only, were positively correlated with disease activity (Kawashima et al., 2022; Khashper et al., 2022), considering that splenic volume was positively affected by BMI and body weight (Harris et al., 2010). To sum up, splenic volume may be a promising indicator of the inflammatory activity of CD. However, the association between splenic volume and IFX treatment in CD remains unclear. Given the fact that IFX therapy altered inflammatory cytokines and adjusted immune response in CD patients, we propose that splenic volume could be affected by IFX treatment and that splenic volume could be a promising indicator to evaluate the efficacy of IFX treatment. Therefore, we expertly analyzed the splenic volume using semi-auto spleen segmentation software. Our goal is to test the hypothesis that splenic volume could be an index to assess the efficacy of IFX treatment in CD patients.

## 2 Materials and methods

### 2.1 Study population

This was a retrospective, single-center, self-control study to assess changes in splenic volume before and after IFX treatment in CD patients. We reviewed the medical records in the Crohn’s disease database of Zhongshan Hospital, Fudan University, between August 2015 and December 2021. Patients aged 18 to 70 years were included in the study if they 1) were diagnosed with CD and received regular IFX therapy in Zhongshan Hospital and 2) received computed tomography (CT) scans within 1 year before IFX therapy and approximately after six sessions of therapy. Patients were excluded if they had 1) exposure to other biologicals (like adalimumab, ustekinumab, and vedolizumab); 2) factors that affected the spleen volume, comprising liver disease, portal hypertension, heart disease, blood disease, infection, and cancer; and 3) a history of splenectomy and other spleen surgery.

Baseline characteristics including sex, age at first IFX therapy, disease duration, BMI, disease location and behavior, presence of extraintestinal manifestations, concomitant drugs, history of bowel surgery, and smoking were recorded. Clinical features including splenic volume, leukocyte and platelet counts, levels of hemoglobin, high-sensitivity C-reactive protein (CRP), erythrocyte sedimentation rate (ESR), albumin, TNF-α, and fecal calprotectin (FCP) were assessed at baseline and after IFX treatment.

The follow-up period ended after an average of six IFX therapy sessions, followed by the measurement of IFX response. The study population was divided into two groups according to IFX response. The response group was defined as remission of symptoms and improvement of clinical indexes after IFX treatment. Otherwise, the patient was classified as the non-response group.

### 2.2 Spleen volume measurement

**FIGURE 1:**
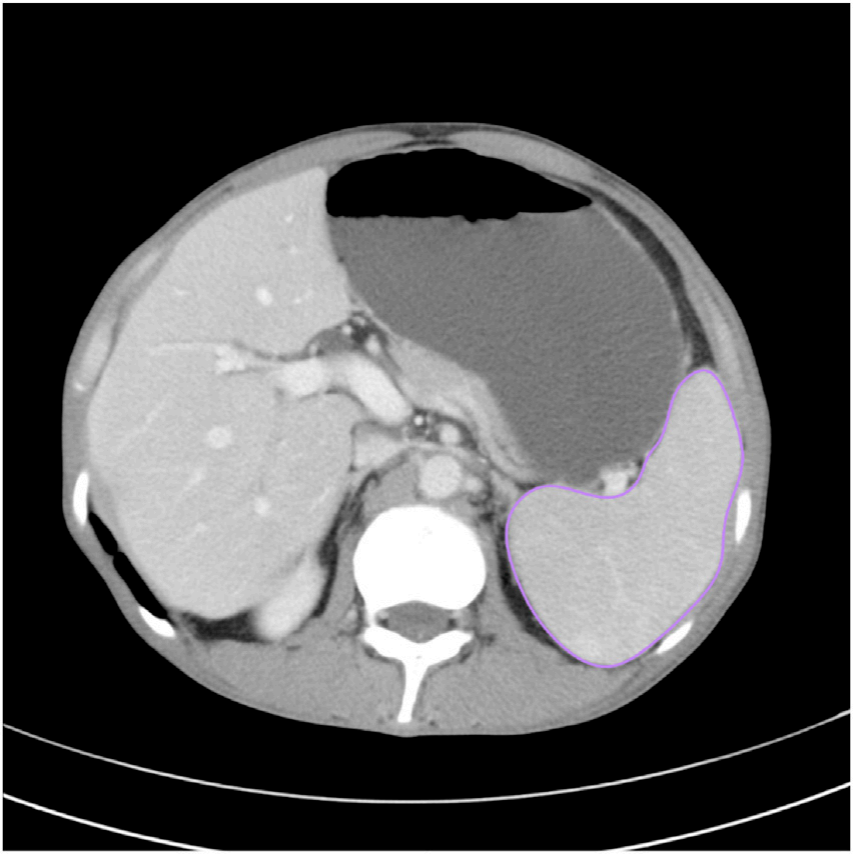
Spleen region of interest (ROI) on the CT slices to measure the splenic volume.

CT scans were performed on SOMATOM Definition AS (Siemens, Erlangen, Germany) or Toshiba Aquilion ONE (Toshiba Medical Systems, Tokyo, Japan) with a slice thickness of 1–3 mm. All CT images were analyzed using semi-auto spleen segmentation software (MM Research Frontier Syngo-Via, VB20, Siemens Healthineers, Germany). Two abdominal radiologists with 6 and 8 years of experience in abdominal imaging manually verified the spleen region of interest (ROI) on each slice to avoid discrepancy. The total spleen volume was calculated by multiplying the overall ROI area with slice thickness (Figure 1).

### 2.3 Statistical analysis

All data were analyzed using IBM SPSS Statistics V26 (SPSS Inc., Chicago, IL, United States) or GraphPad Prism 9 (GraphPad Software, San Diego, CA, United States). Continuous variables were presented as median with standard deviation (SD) and categorical variables as the frequency with percentages. Quantitative variables were compared using a *t*-test. Categorical variables were analyzed by Fisher’s exact test. Pearson’s correlation coefficient was used to evaluate the relationship between the clinical parameters and BMI-adjusted splenic volume in all patients. A two-tailed *p* < 0.05 represented statistical significance.

### 2.4 Ethical consideration

Informed consent was obtained from all individuals. The study was approved by the Ethics Committee of Zhongshan Hospital, Fudan University (No. B2023-093).

## 3 Results

### 3.1 Characteristics of patients

A total of 49 patients with CD who received regular IFX treatment at Zhongshan Hospital, Fudan University, between August 2015 and December 2021 were included in this retrospective cohort study. The disease characteristics and clinical parameters at baseline of the study population are shown in Table 1; Supplementary Table S1, respectively. These patients were new users of IFX, of which 41 were responsive to IFX, while 8 were non-responsive after an average of six therapy sessions. Overall, the response group seemed slightly younger at initial IFX therapy than the non-response group (31.3 ± 11.5 vs. 51.5 ± 7.3 years), although without any significant difference. The mean disease duration before biologic initiation (3.6 ± 4.0 vs. 4.2 ± 2.1 years), average BMI (19.0 ± 3.6 vs. 19.8 ± 4.2 kg/m^2^), and the body weight (55.8 ± 11.1 vs. 56.9 ± 17.5 kg) showed no significant difference. The two groups indicated different disease locations. The non-response group was more likely to be exposed to glucocorticoid and thalidomide before biologic initiation. Among all clinical parameters, only the serum albumin level (*p* < .01) was significantly different between the two groups (Supplementary Table S1).

**TABLE 1:** Disease characteristics at baseline.

	Response patient (*n* = 41)	Non-response patient (*n* = 8)	*p*
Male	29	5	0.11
Age at first infliximab therapy (years)	31.3 ± 11.5	51.5 ± 7.3	0.06
Disease duration (years)	3.6 ± 4.0	4.2 ± 2.1	0.12
Body mass index (kg/m^2^)	19.0 ± 3.6	19.8 ± 4.2	0.91
Body weight (kg)	55.8 ± 11.1	56.9 ± 17.5	0.82
Disease location			<0.05
L1 (ileal disease)	13 (31.7)	6 (75.0)	
L2 (colonic disease)	1 (2.4)	1 (12.5)	
L3 (ileocolonic disease)	25 (61.0)	1 (12.5)	
Presence of upper GI disease	2 (4.9)	0 (0)	
Disease behavior			0.38
B1 (non-stricturing and non-penetrating)	25 (61.0)	5 (62.5)	
B2 (stricturing)	12 (29.3)	1 (12.5)	
B3 (penetrating)	4 (9.8)	2 (25)	
Perianal disease	20	1	0.12
Presence of extraintestinal manifestations	2	0	1
Concomitant			
5-Aminosalicylic acid	20	5	0.7
Glucocorticoid	13	8	<0.001
Azathioprine	12	5	0.11
Methotrexate	4	2	0.25
Cyclosporin A	0	1	0.16
Thalidomide	2	2	<0.01
Previous bowel surgery	14	1	0.41
History of smoking	3	0	1

Proportions are reported as no. (%). Continuous variables are reported as mean ± SD. *p* represents the *p*-value.

### 3.2 Distinct clinical parameters and splenic volume after IFX treatment

In patients responsive to IFX, splenic volume, as well as volume adjusted for BMI (SV/BMI) and body weight (SV/W), significantly decreased after IFX treatment compared to the volume before the treatment (*p* < .001), illustrating the correlation between splenic volume and IFX efficacy. Moreover, effective IFX treatment contributed to the upregulated levels of hemoglobin and albumin (*p* < .0001), which demonstrated improvement in the disease. On the contrary, IFX treatment led to downregulated levels of leukocyte count (*p* < .01), platelet count (*p* < .001), CRP (*p* < .001), and ESR (*p* < .01), which were positively correlated with disease activity. TNF-α also showed a discrepant level after biological exposure (*p* < .0001), while FCP was not significantly different (Table 2).

**TABLE 2:** Comparison of clinical parameters and splenic volume before and after infliximab treatment in response patients.

	Before treatment	After treatment	*p*	n
Splenic volume (cm^3)	248.4 ± 101.7	232.0 ± 88.2	0.00057	41
SV/BMI (cm^5*10^4/kg)	13.5 ± 5.8	11.5 ± 4.7	9.11E-07	41
SV/W (cm^3/kg)	4.5 ± 1.8	3.9 ± 1.4	1.00E-06	41
Hemoglobin (g/L)	114.8 ± 23.3	133.6 ± 17.9	3.22E-07	41
Leukocyte count (×10^12/L)	7.6 ± 3.3	5.6 ± 1.7	0.0034	41
Platelet count (×10^9/L)	330.7 ± 118.1	257.4 ± 64.4	0.00011	41
C-reactive protein (mg/L)	34.8 ± 43.3	9.0 ± 15.6	0.00049	41
Erythrocyte sedimentation rate (mm/h)	32.0 ± 24.6	19.8 ± 20.7	0.0078	41
Albumin (g/L)	37.8 ± 6.7	44.5 ± 4.5	1.30E-08	41
TNF-α (pg/mL)	16.2 ± 17.8	148.0 ± 127.2	0.000050	23
Fecal calprotectin (μg/g)	362.2 ± 94.0	303.8 ± 317.0	0.46	10

SV/BMI, splenic volume adjusted by body mass index; SV/W, splenic volume adjusted by body weight; TNF-α, tumor necrosis factor-α; *p* represents the *p*-value; n represents the number of cases.

To further verify whether IFX affected the splenic volume, we observed the alteration in splenic volume in the non-response group. Conversely, splenic volume, SV/BMI, and SV/W were increased after IFX treatment (*p* < .01). None of the blood and fecal indicators were significantly different (Table 3).

**TABLE 3:** Comparison of clinical parameters and splenic volume before and after infliximab treatment in non-response patients.

	Before treatment	After treatment	*p*	n
Splenic volume (cm^3)	186.6 ± 57.3	234.8 ± 50.9	0.0069	8
SV/BMI (cm^5*10^4/kg)	9.7 ± 3.5	13.1 ± 4.1	0.0032	8
SV/W (cm^3/kg)	3.5 ± 1.5	4.8 ± 1.9	0.0039	8
Hemoglobin (g/L)	113.6 ± 25.9	124.4 ± 24.4	0.49	8
Leukocyte count (×10^12/L)	7.1 ± 2.8	7.2 ± 2.9	0.92	8
Platelet count (×10^9/L)	308.4 ± 161.9	278.3 ± 95.8	0.42	8
C-reactive protein (mg/L)	34.1 ± 43.6	60.3 ± 89.3	0.24	8
Erythrocyte sedimentation rate (mm/h)	28.4 ± 16.4	33.3 ± 18.4	0.42	8
Albumin (g/L)	38.8 ± 1.8	39.4 ± 3.5	0.73	8
TNF-α (pg/mL)	29.3 ± 26.2	107.4 ± 116.3	0.22	5
Fecal calprotectin (μg/g)	245.3 ± 259.6	367 ± 492.3	0.59	2

SV/BMI, splenic volume adjusted by body mass index; SV/W, splenic volume adjusted by body weight; TNF-α, tumor necrosis factor-α; *p* represents the *p*-value; n represents the number of cases.

### 3.3 The correlation between clinical parameters and splenic volume before IFX treatment

**FIGURE 2:**
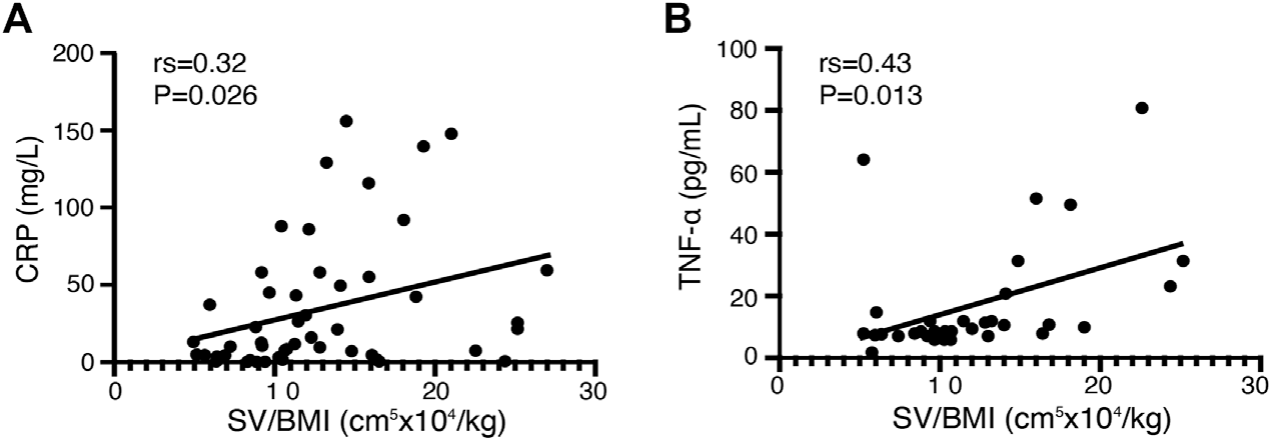
Correlation between clinical parameters and SV/BMI in all CD patients before infliximab treatment. **(A)** The C-reactive protein (CRP) level in patients with CD before infliximab treatment shows a significant positive correlation with SV/BMI (*n* = 49). **(B)** The tumor necrosis factor (TNF)-α level in patients with CD before infliximab treatment shows a significant positive correlation with SV/BMI (*n* = 28).

We conducted correlation analysis in both response and non-response patients included in our study before infliximab treatment to investigate the association between clinical indicators and splenic volume. Both CRP and TNF-α levels showed a significantly positive correlation with the splenic volume adjusted for BMI (rs = 0.32, *p* < .05, *n* = 49 and rs = 0.43, *p* < .05, *n* = 28, respectively; Figure 2). No significant correlations were found in other clinical parameters, including hemoglobin level, leukocyte count, platelet count, ESR level, albumin level, and FCP level.

## 4 Discussion

IFX is one of the crucial biologicals in the treatment of CD. However, there is still a lack of effective indicators for IFX therapeutic response. Splenomegaly in CD is already well described previously, while few studies explained the role of splenic volume in IFX treatment response of CD patients. We aimed to investigate whether the splenic volume can be a new biomarker in evaluating the therapeutic efficacy of IFX in patients with CD.

Uni Wong and Raymond K. Cross summarized the risk factors for primary non-response to IFX, involving high BMI, high TNF-α level, and small bowel involvement, among which high TNF-α concentration may associate with high inflammatory burden and cause low efficacy of IFX (Olsen et al., 2009; Wong and Cross, 2017). In addition, a higher level of TNF-α was discovered in non-responders with fistulizing CD than in responders before IFX treatment (Martínez-Borra et al., 2002). In our study, patients in the non-response group showed slightly higher levels of BMI and TNF-α and a higher percentage of ileal disease than those in the response group, although the first two differences failed to reach statistical significance. These factors seemed to contribute to the occurrence of loss of response to IFX in the non-response group.

In addition, non-responders were more likely to accept glucocorticoid and thalidomide before biological initiation (Table 1). Glucocorticoid is one of the primary pharmaceuticals to enter or maintain disease remission. When an adequate dosage of glucocorticoid is administered to CD patients but still failed to reach disease remission, it implies that these patients are refractory and more likely correlated with a lack of response to IFX. Thalidomide plays a role in the treatment of refractory CD through mediating TNF-α reduction, T-cell regulation, angiogenesis inhibition, and other (non-)immunomodulatory mechanisms (Ginsburg et al., 2001). These therapeutic properties are limited by its potential adverse effects. Patients who receive thalidomide represent serious cases where non-response to IFX may occur.

Splenic volume is known to enlarge in CD patients compared to a healthy population, especially in the active stage (Kawashima et al., 2022; Khashper et al., 2022). Few studies focus on the influence of IFX treatment on splenic volume in CD. Our study illustrated that IFX treatment recovered the splenic volume in responders (Table 2), while the mechanism remains unclear. The spleen is known to not only act as a phagocytic filter but also play an essential role in regulating immune homeostasis and protecting against infections by encapsulated bacteria (Di Sabatino et al., 2013). TNF controls various homeostatic chemokines, including CXCL13, CCL19, and CCL21, thus maintaining correct organization in the white-pulp region of the spleen (Mebius and Kraal, 2005). A previous study revealed that IFX treatment restored splenic function in responders rather than in non-responders as pitted red cell values decreased, accompanied by a parallel increase in the IgM-memory B-cell pool, which survived or generated depending on the spleen (Di Sabatino et al., 2008). The complex mechanism of how IFX adjusts the splenic volume in responders may lie in the reduction of the disease inflammatory burden and regulation of the spleen-related immune system. On the contrary, the increased splenic volume after IFX therapy in non-responders may be a result of disease progression (Table 3).

In some patients, CD is accompanied by anemia and hypoproteinemia, which can be relieved by valid therapies (Lönnkvist et al., 2009). In our study, restoration of hemoglobin and albumin levels in responders indicated that the IFX treatment is effective (Table 2).

CRP, an acute-phase reactant produced by the liver in response to circulating inflammatory cytokines, including TNF-α, is related to mucosal inflammation in CD patients, which can be rapidly reduced as a result of biological therapy (Levin et al., 2016). Consistently, platelets actively contribute to mucosal inflammation and injury in IBD and are notably elevated in active CD (Kapsoritakis et al., 2001; Danese et al., 2003). Additionally, leukocytes and ESR are also known as inflammatory indicators in CD (Tang et al., 2020). Given the aforementioned facts, we observed a reduction in these serum parameters as a result of IFX treatment in responders (Table 2), which revealed that IFX played a role in inducing and maintaining disease remission.

A previous study demonstrated a significant increase in the TNF-α level after 6 weeks of IFX treatment in both sustained response and non-sustained response groups (Ogawa et al., 2012). Accordingly, the TNF-α level was significantly upregulated in the response group (Table 2), and a trend to ascend was observed in the non-response group after IFX administration in our study (Table 3). This phenomenon was also described in rheumatoid arthritis and juvenile idiopathic arthritis (Charles et al., 1999; Levälampi et al., 2007). However, the underlying mechanism remains to be well understood.

TNF-α is an important proinflammatory cytokine in CD (Tang et al., 2020). A previous study figured out that the TNF-α level was higher in active CD compared to the disease in the remission stage (Ogawa et al., 2012), which revealed that TNF-α, as same as CRP, is positively associated with CD activity. In our study, SV/BMI showed a positive correlation with both TNF-α and CRP at baseline (Figure 2); hence, SV/BMI can partly indicate disease activity.

Harris et al. (2010) verified that splenic volume was negatively correlated with age and positively correlated with body weight, BMI, and body surface area (BSA). In previous studies, splenic volume was adjusted by dividing by either body weight or BMI (Kawashima et al., 2022; Khashper et al., 2022). In our study, although splenic volume, SV/BMI, and SV/W all showed significant differences before and after IFX treatment in both groups, SV/BMI provided the strongest significance among these indicators as its *p*-value was the smallest (SV *p* = 0.00057, SV/BMI *p* = 9.11E-07, and SV/W *p* = 1.00E-06 in the response group), which suggested that SV/BMI may be a better indicator (Table. 2). However, taking age and BSA into account and finding a rational method to modulate splenic volume may help us figure out an accurate indicator.

Splenic volume, calculated based on CT examination, is non-invasive, convenient, and costless. However, splenic volume can be affected by opportunistic infections and portal hypertension related to IBD (Talbot et al., 1986; Maconi et al., 2012). Considering the novel role of the splenic volume in evaluating IFX efficacy, it is worth investigating its predictive value in future research. In our study, the splenic volume, SV/BMI, and SV/W failed to reach statistical significance before IFX treatment between the two groups as a result of the small sample size of the non-response group (Supplementary Table S1). We should further conduct a study with enlarged sample size and strict exclusion criteria so as to explore the predictive role of splenic volume, as well as build a prediction model of infliximab efficacy.

There were some limitations to the study. First, this is a retrospective design with a small sample size and a non-double-blind setup. The sample size of the non-response group was not big enough to lay out significant differences in splenic volume and other clinical features between responders and non-responders before IFX treatment, except for the albumin level. The regular follow-up CT examination after IFX therapy was not conducted in many CD patients. In addition, several CD patients received MRI of the small intestinal before IFX therapy, which did not scan the spleen completely and could not be used to measure splenic volume. These two factors both accounted for the small sample of the research. Second, inherent to a retrospective attribute, some of the clinical features were lost. Consequently, FCP failed to show significant differences before and after IFX treatment in responders. In some CD cases, the data on the TNF-α level were lost. Additionally, CDAI failed to be accurately calculated, and the relationship between disease activity and splenic volume was not well proved.

## 5 Conclusion

In conclusion, we performed a retrospective study based on the CD database of Zhongshan Hospital and an accurate method to measure splenic volume in patients with CD. We revealed that the splenic volume, especially volume adjusted for BMI and body weight, was reduced after effective IFX treatment, and the splenic volume adjusted for BMI was positively correlated with disease activity. In a word, splenic volume can be an emerging indicator to valuate IFX efficacy and serve personalized treatment in CD.

## Data Availability

The data underlying this article can be found in online repositories: https://ngdc.cncb.ac.cn/omix/, OMIX ID: OMIX003866, further inquiries can be directed to the corresponding authors.

## Abbreviations

CD, Crohn’s disease; IBD, inflammatory bowel disease; IFX, infliximab; BMI, body mass index; SV/W, splenic volume adjusted by body weight; SV/BMI, splenic volume adjusted by BMI; CRP, C-reactive protein; ESR, erythrocyte sedimentation rate; TNF-α, tumor necrosis factor-α; CT, computed tomography; FCP, fecal calprotectin; ROI, region of interest; SD, standard deviation; BSA, body surface area.
