# Supplementary material for: A novel role of the splenic volume in Crohn’s disease: evaluating the efficacy of infliximab
Source: Front Pharmacol. 2023 Aug 17;14:1246657. doi: 10.3389/fphar.2023.1246657 (PMC10470019; doi:10.3389/fphar.2023.1246657)
Supplement: Supplementary file 1 [file Table1.DOCX]

Supplementary Table S1. Clinical Parameters at Baseline

|  | response patients (n=41) | non-response patients (n=8) | P |
| --- | --- | --- | --- |
| Splenic volume (cm^3) | 248.4±101.7 | 186.6±57.3 | 0.28 |
| SV/BMI (cm^5*10^4/kg) | 13.5±5.8 | 9.7±3.5 | 0.15 |
| SV/W (cm^3/kg) | 4.5±1.8 | 3.5±1.5 | 0.45 |
| Hemoglobin (g/L) | 114.8±23.3 | 113.6±25.9 | 0.60 |
| Leukocyte count (×10^12/L) | 7.6±3.3 | 7.1±2.8 | 0.51 |
| Platelet count (×10^9/L) | 330.7±118.1 | 308.4±161.9 | 0.56 |
| C-reactive protein (mg/L) | 34.8±43.3 | 34.1±43.6 | 0.91 |
| Erythrocyte sedimentation rate (mm/h) | 32.0±24.6 | 28.4±16.4 | 0.18 |
| Albumin (g/L) | 37.8±6.7 | 38.8±1.8 | <0.01 |
| TNF-α (pg/mL) | 16.2±17.8 | 29.3±26.2 | 0.05 |
| Fecal calprotectin (μg/g) | 362.2±94.0 | 245.3±259.6 | 0.52 |

SV/BMI, splenic volume adjusted by body mass index; SV/W, splenic volume adjusted by body weight; TNF-α, albumin and tumor necrosis factor-α. Continuous variables reported as mean ± SD. P represents P value.
